# Age-Related Regional Changes in Choroidal Vascularity in Healthy Emmetropic Eyes

**DOI:** 10.1167/tvst.14.5.3

**Published:** 2025-05-01

**Authors:** Ghazal Valizadeh, Hosein Hoseini-Yazdi, Scott Read, David Alonso-Caneiro, Michael Collins

**Affiliations:** 1 Save Sight Institute, Discipline of Ophthalmology, The University of Sydney, Sydney, New South Wales, Australia; 2 Queensland University of Technology (QUT), Contact Lens and Visual Optics Laboratory, Centre for Vision and Eye Research, Optometry and Vision Science, Kelvin Grove, Queensland, Australia; 3 School of Science, Technology and Engineering, University of Sunshine Coast, Petrie, Queensland, Australia

**Keywords:** aging, choroid, optical coherence tomography

## Abstract

**Purpose:**

This retrospective cross-sectional study examined regional changes in choroidal vascularity index (CVI) with physiological aging in healthy emmetropes.

**Methods:**

Deep learning methods were used for segmentation and binarization of enhanced depth imaging optical coherence tomography images of the choroid collected from 280 healthy emmetropic subjects (mean spherical equivalent refraction: +0.39 ± 0.38 D), including 83 children (5–12 years), 77 adolescents (13–17 years), and 120 adults (18–41 years). The CVI, calculated as the ratio of luminal versus total choroidal area (in percent), and luminal and stromal choroidal thickness were measured across the 5-mm horizontal macular region centered on the fovea. Linear mixed models were used to examine age-related regional changes in the choroid while controlling for gender and imaging time of day.

**Results:**

The macular CVI reduced significantly from childhood (65% ± 0.5%) and adolescence (63% ± 0.5%) to adulthood (59% ± 0.4%) (*P* < 0.001). Significant regional variations were observed (*P* < 0.001) with the CVI increasing from the fovea (61% ± 0.3%) toward the perifovea (64% ± 0.3%) and from the temporal (61.4% ± 0.3%) toward the nasal hemiretina (63% ± 0.3%). The age-related decrease in the CVI was greater in the nasal (−7% ± 0.7%) than the temporal (−6% ± 0.7%) macula (*P* = 0.014) and was associated with a significant nasal stromal thickening (45 ± 5 µm; *P* < 0.001) and temporal luminal thinning (−16 ± 6 µm; *P* = 0.033) from childhood to adulthood.

**Conclusions:**

Physiological aging was associated with a significant region-dependent decline in the CVI driven, primarily by stromal thickening in the nasal and luminal thinning in the temporal macula.

**Translational Relevance:**

These age-related changes in the CVI provide new insights into the physiological morphology of the choroid during aging and may aid clinicians in understanding the spatial and age-associated predilections of certain chorioretinal diseases.

## Introduction

The vascular choroid plays a crucial role in providing the outer retina with oxygen and nutrients, is involved in the mechanisms regulating eye growth, and is implicated in the pathophysiology of a variety of chorioretinal diseases.^1^ Detailed in vivo evaluation of the choroid has become possible with the advent of optical coherence tomography (OCT), particularly the use of enhanced depth imaging (EDI) mode, allowing better choroidal imaging and subsequent introduction of novel choroidal biomarkers.^1^^,^^2^ One such parameter measured with OCT is choroidal thickness (ChT), which is widely used as an indicator of ocular and systemic health^2^^–^^4^ and varies with a range of physiological factors, including age,^5^ axial length,^6^ refractive error,^7^ measurement eccentricity,^8^ and time of day.^9^

However, measures of ChT do not differentiate between the stromal and luminal vascular components of the choroid, which may be influenced differently by ocular diseases.^2^ This limitation has been overcome through binarization of OCT images to distinguish the luminal from the stromal components of the tissue, allowing for calculation of the choroidal vascularity index (CVI) as the ratio of luminal area to the total choroidal area.^4^ CVI reflects the choroidal angioarchitecture and provides a non-invasive approach for the diagnosis, monitoring, and prognostication of various ocular diseases, including age-related macular degeneration, central serous chorioretinopathy, diabetic retinopathy, uveitis, and retinal dystrophies.^10^^,^^11^

In studies examining healthy eyes, the CVI is found to vary widely between 40% and 70%.^4^^,^^5^^,^^12^^–^^21^ Associations between the CVI and factors such as age, gender, retinal region, and axial length have been reported, although sometimes with conflicting outcomes.^4^^,^^13^^,^^14^ Whereas some studies have found no significant correlation between CVI and age,^22^^,^^23^ or a positive correlation,^20^^,^^24^ most have found a negative association, consistent with a decrease in luminal to total choroidal area with age.^12^^,^^14^^,^^15^^,^^25^

Both ChT and the CVI have been shown to vary depending on the region of measurement across the retina.^8^^,^^26^ The choroid is thickest at the fovea and subsequently thins toward the periphery, with nasal and inferior peripheral regions typically reported to show the greatest degree of thinning.^8^^,^^27^^,^^28^ In contrast, the literature on regional changes in the CVI is less consistent. Although some studies have found a decrease in the CVI with increasing eccentricity,^14^ others have found the inverse to be true.^8^^,^^26^ Changes in the CVI across the retinal quadrants have also been described, although again with variable results.^8^^,^^14^^,^^26^

Overall, existing literature on the relationship among the CVI, age, and retinal region remains conflicting and nuanced. The aim of this study was to examine the physiological age-related changes and regional variations in the CVI in a cohort of emmetropic participants, utilizing automated image analysis tools. As age and choroidal vascularity are both key factors in the development of many chorioretinal diseases, understanding the normal age-related changes in choroidal angioarchitecture is fundamental to our knowledge of chorioretinal pathophysiology.

## Methods

### Subjects

In this retrospective cohort study, data from 280 healthy individuals, between the ages of 5 and 41 years, were included for analysis. These participants had previously taken part in various research studies at the Queensland University of Technology that were approved by the Queensland University of Technology Human Research Ethics Committee and conducted in accordance with the tenets of the Declaration of Helsinki. Written informed consent was obtained from all participants prior to study participation.

Participants’ eligibility was assessed based on detailed history and ocular examinations, including non-cycloplegic subjective refraction, slit-lamp examination, ocular biometry, and OCT. Only one eye from each participant was analyzed. The inclusion criteria consisted of healthy non-smoking participants with no significant history of ocular or systemic diseases. No participants were pregnant. Only emmetropic participants were included with spherical equivalent refractive error from −0.5 D to +1 D and astigmatism of less than −1.5 D based on subjective refraction, with visual acuity of 0.00 logMAR or better in each eye. All adult participants (greater than 18 years) were asked to refrain from the consumption of alcohol and coffee for a minimum of 4 hours prior to each study session. Exclusion criteria involved history or evidence of any ocular or systemic pathology, use of any ocular medications, and subjects with poor-quality OCT images with non-identifiable chorioscleral interface or scans not optimally centered on the fovea.

All measurements were taken from 8:30 AM to 5:30 PM. To minimize the impact of prior near visual activities on the choroidal measurements, OCT imaging was performed following a minimum 10-minute wash-out period of distance viewing.^29^^,^^30^ Constant low photopic lighting conditions were maintained for the duration of the wash-out period and choroidal imaging (∼10 lux) to minimize the effect of variations in light exposure on ChT.^31^ Measurements were taken without mydriasis to avoid potential changes in ChT and vasculature associated with pharmacological agents.^32^

### Procedures

OCT imaging and ocular biometry were performed on all subjects using the Heidelberg SPECTRALIS spectral-domain OCT instrument (Heidelberg Engineering, Heidelberg, Germany) and Lenstar LS 900 optical biometer (Haag-Streit, Köniz, Switzerland). All OCT images were acquired from the central 5-mm macular region using a high-resolution horizontal line scan centered on the fovea with the application of EDI mode to optimize the visibility of the choroid. The automatic real-time eye tracking feature of the instrument was used to average a minimum of 30 B-scans, thereby reducing speckle noise, enhancing image contrast, and optimizing choroidal visibility.^33^ The OCT data analyzed in this study was sourced from previous studies, which have reported coefficients of repeatability for repeated measures of choroidal thickness in the macular region ranging from 6 to 16 µm, indicating excellent repeatability.^29^^,^^33^^–^^38^ Choroidal imaging was repeated three times for each participant, and the best quality scan was selected for CVI analysis, with a mean quality index score of 34.6 ± 4.8 dB for all included OCT scans.

### Image Analysis

**Figure 1.**
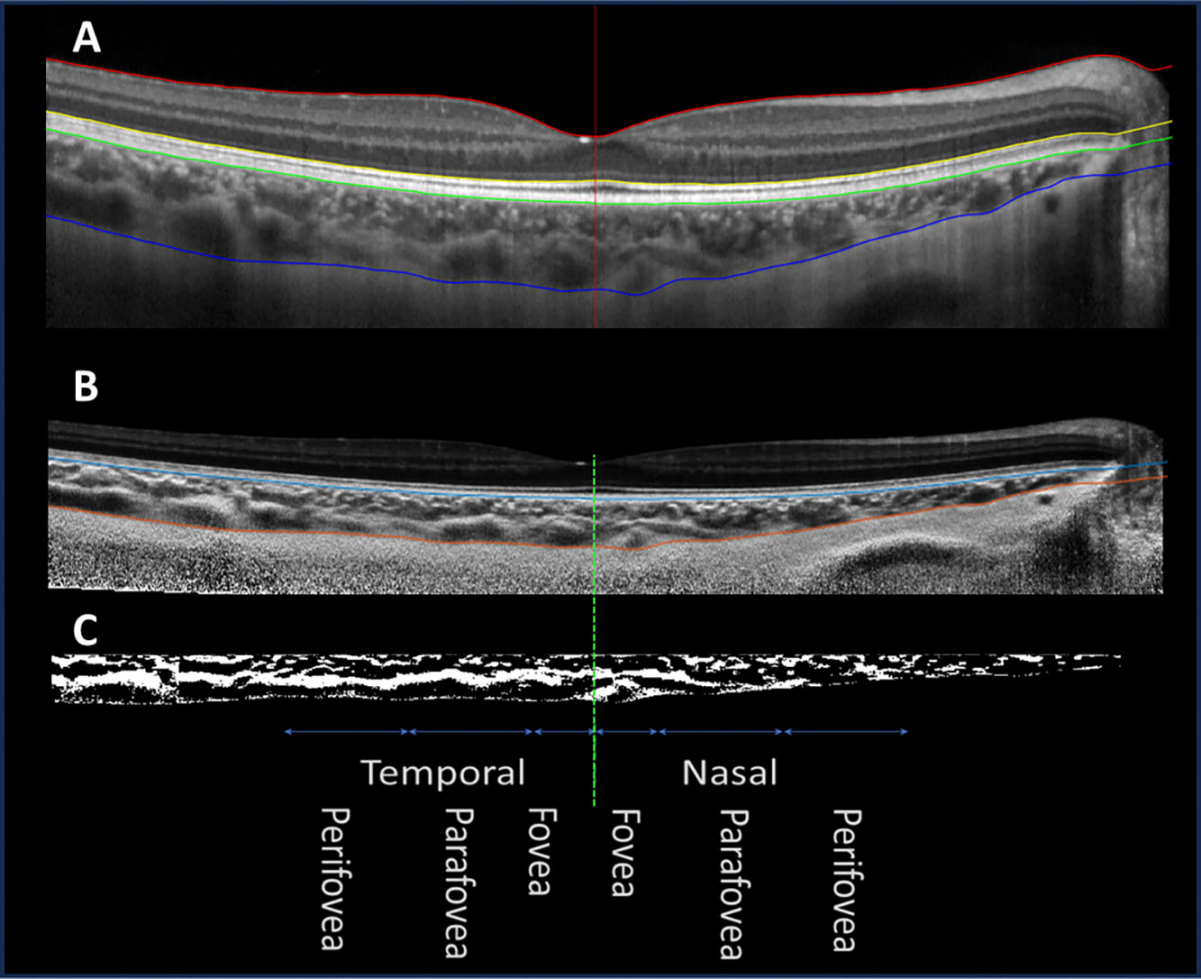
Example of OCT image segmentation and binarization of the choroid in a representative B-scan along the horizontal meridian. (**A**) Choroid segmentation where the *green line* demarcates the inner choroidal boundary of the retinal pigment epithelium/Bruch's membrane complex, and the *blue line* represents the outer choroidal boundary at the chorioscleral interface. The *vertical red line* marks the position of the fovea at the foveal pit. (**A**) Each segmented OCT scan was then flattened (**B**) before undergoing binarization within the choroidal region of interest (**C**), where the *black pixels* represent choroidal luminal area, and the *white pixels* signify choroidal stromal area. The *green dotted line* demarcates the nasal and temporal macula hemiretina. Three macula eccentricities were defined for each hemiretina as the fovea (0–500 µm), parafovea (500–1500 µm), and perifovea (1500–2500 µm).

Choroidal boundaries were automatically segmented for each B-scan image using deep learning algorithms that have been described in detail and validated in a number of previous publications.^39^^,^^40^ The anterior choroidal boundary was defined as the outer surface of the hyper-reflective line coinciding with retinal pigment epithelium/Bruch's membrane complex, whereas the outer choroidal boundary was represented by the inner surface of the hyper-reflective line demarcating the chorioscleral interface (Fig. 1A). All automatically segmented B-scans were reviewed by an experienced masked observer for segmentation errors and manually corrected if needed. The deepest point in the foveal pit was manually marked in each OCT image, corresponding to the position of the fovea, and demarcated the border between the nasal and temporal hemiretina.

### Choroidal Thickness Calculation

ChT measurements were calculated as the axial distance (i.e., along the A-scan) between the anterior and posterior boundaries of the choroid across the central 5 mm of each scan. ChT measurements were averaged across the foveal (central 1-mm diameter), parafoveal (1- to 3-mm diameter zone), and perifoveal (3- to 5-mm diameter zone) macular eccentricities and the nasal and temporal macula (Fig. 1C). All images were analyzed as if for the right eye to account for the regional anatomical variations between the right and left eyes associated with the position of the optic nerve head. In cases of B-scans of the left eye, the image was flipped along the horizontal dimension.

### Choroidal Vascularity Index Calculation

Each segmented B-scan was flattened (Fig. 1B) and then automatically binarized using custom-written software based on deep learning algorithms, which avoids the bias of a constant local binarization window size typically used in previous methods (Fig. 1C), the details of which have been previously published.^41^ The deep learning algorithms ensure that the image binarization captures well both large choroidal stromal and luminal components while also maintaining the small vasculature details. CVI and ChT measurements were examined across the macular regions, centered on the fovea, after taking into account the lateral image magnification caused by variations in the refractive error and ocular biometry.^34^ Within the binarized B-scans, the luminal and stromal choroid are represented by black and white pixels, respectively (Fig. 1C). The CVI was calculated as the ratio of luminal area to total choroidal area (in percentage). The calculated CVI was then averaged across each macula eccentricity and hemiretina for all B-scans. Luminal thickness was calculated as CVI × ChT and stromal thickness as ChT – luminal thickness (in microns).

### Statistical Analysis

Participants were categorized into three groups according to age, including children between 5 and 12 years, adolescents between 13 and 17 years, and adults between 18 and 41 years. All statistical analyses were performed using SPSS Statistics 29 (IBM, Chicago, IL). Normality of data for each outcome measurement was confirmed using the Kolmogorov–Smirnov test. Gender distribution among the age groups was compared using the χ^2^ test. Linear mixed model (LMM) analyses were performed separately for each choroidal parameter including ChT, CVI, luminal thickness, and stromal thickness. In each LMM, the hemiretina (nasal, temporal), eccentricity (fovea, parafovea, perifovea), age group (child, adolescent, adult), gender, and all possible interactions were included as fixed factors. Because an analysis of variance found a significant variation in time of imaging across different age groups (*P* < 0.001), the effect of time of imaging was also adjusted for as a continuous variable in each LMM. Slope and intercept of individual participants were considered as random factors in each model using a variance components covariance structure. Univariate and multivariate linear regression analyses were performed to examine the association among age (as a continuous variable), ChT, and axial length, with each choroidal vascularity parameter averaged across the macula. The influence of axial length, gender, and imaging time of day was also adjusted for in the multivariate analyses. Bonferroni correction was applied for post hoc comparisons and *P* < 0.05 was considered statistically significant. Outcome measurements are reported as mean ± standard error of the mean (SEM).

## Results

### Subject Demographics

The mean age of participants was 18 ± 8 years (range, 5–41), with 155 participants (55%) being female (Table 1). Gender distribution was similar among the three age groups (*P* = 0.083). Of the participants, 215 were Caucasian (76.8%) and 65 were non-Caucasian (23.2%). There was a significant variation in the ethnicity distribution across age groups (*P* < 0.001). The majority of children and adolescents were Caucasian (95% and 92%, respectively), whereas, among adults, the proportion of Caucasian participants (54%) was comparable to non-Caucasian participants (46%). All subjects were emmetropic with a mean spherical equivalent refraction of +0.39 ± 0.38 D and a mean axial length of 23.35 ± 0.71 mm.

**Table 1.** Participant Demographics for Each Age Group (*N* = 280; 155 Females, 125 Males)

	Mean ± SD (Range)			
	Children (*n* = 83)	Adolescents (*n* = 77)	Adults (*n* = 120)	Total (*n* = 280)
Spherical equivalent refraction (D)	0.51 ± 0.35 (−0.38 to 1)	0.46 ± 0.36 (−0.5 to 1)	0.27 ± 0.37 (−0.5 to 1)	0.39 ± 0.38 (−0.5 to 1)
Age (y)	10 ± 2 (5–12)	14 ± 1 (13–17)	26 ± 7 (18–41)	18 ± 8 (5–41)
Axial length (mm)	23.14 ± 0.63 (21.71–24.78)	23.34 ± 0.71 (21.57–25.26)	23.49 ± 0.72 (21.83–25.68)	23.35 ± 0.71 (21.57–25.68)
Macular ChT (µm)	317 ± 65 (214–483)	343 ± 67 (200–481)	349 ± 64 (179–537)	338 ± 67 (179–537)
Macular CVI (%)	65.0 ± 4.8 (52.8–75.6)	63.1 ± 4.3 (51.9–74.4)	58.8 ± 3.9 (51.8–70.65)	61.8 ± 5.1 (51.8–75.6)
Macular luminal thickness (µm)	204 ± 37 (136–289)	215 ± 43 (131–312)	204 ± 36 (109–292)	207 ± 39 (109–312)
Macular stromal thickness (µm)	113 ± 34 (58–214)	127 ± 30 (59–197)	144 ± 32 (69–252)	130 ± 35 (58–252)

### Choroidal Thickness 

**Figure 2.**
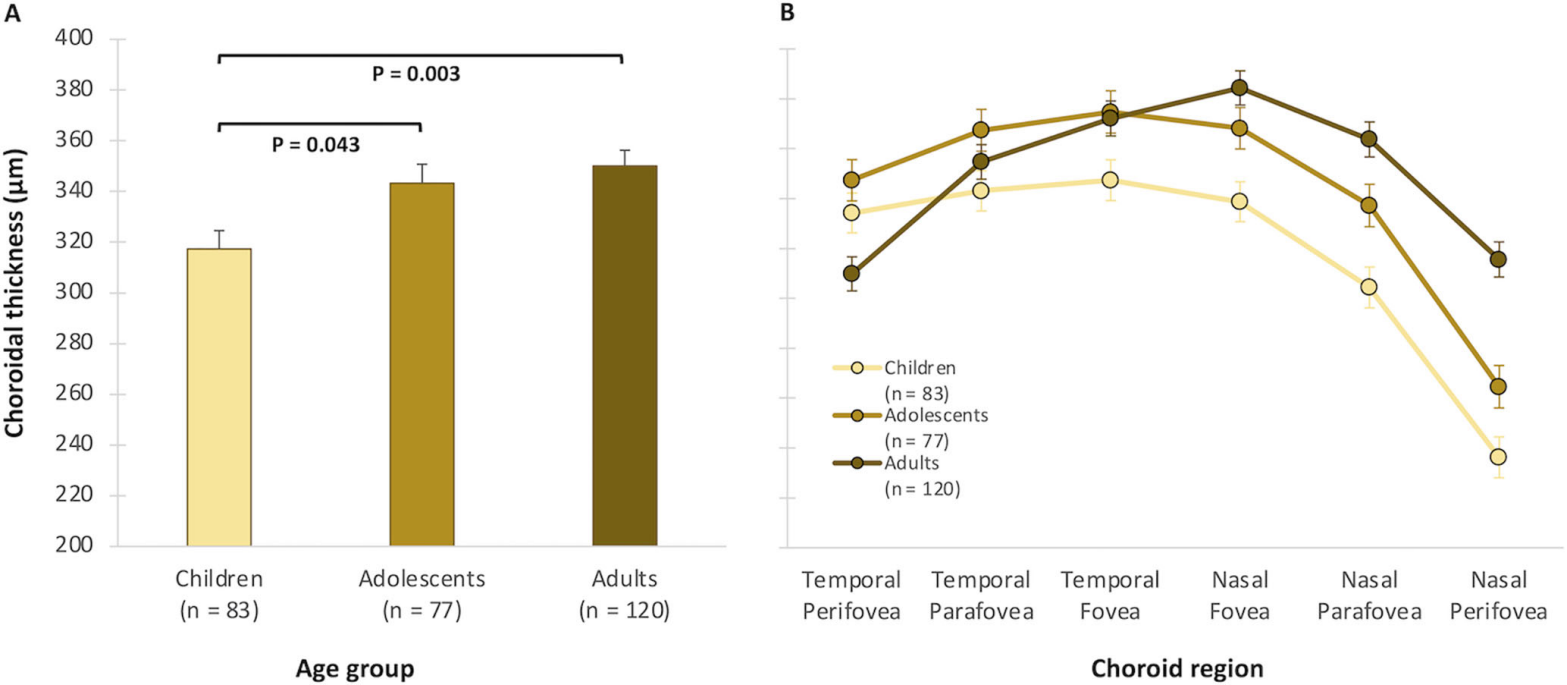
(**A**) Mean macular ChT for each age group adjusted for gender and imaging time of day. (**B**) Regional ChT for each age group across different macular eccentricities and hemiretina. *Error bars* indicate the SEM.

Overall, the mean macular ChT was 338 ± 67 µm. ChT did not vary significantly with gender or by the imaging time of day (both *P* > 0.05). However, there was a significant main effect of age (*P* = 0.003), with a significant increase in ChT observed from childhood (317 ± 7 µm) to adolescence (343 ± 8 µm; *P* = 0.043) but not from adolescence into adulthood (350 ± 6 µm; *P* = 0.99) (Fig. 2). ChT varied significantly with eccentricity (*P* < 0.001), exhibiting its greatest thickness at the fovea (364 ± 4 µm), and it progressively thinned toward the parafovea (345 ± 4 µm) and perifovea (301 ± 4 µm). However, the eccentricity by age interaction was not statistically significant (*P* = 0.479) (Table 2). ChT also varied significantly between the nasal macula (324 ± 4 µm) and temporal macula (350 ± 4 µm; *P* < 0.001). A significant hemiretina by age interaction was also observed (*P* < 0.001), with the ChT increasing with age only in the nasal but not in the temporal macula. There was a significant hemiretina by age by gender interaction (*P* = 0.003) whereby the age-related increase in nasal ChT was observed at an earlier age in females compared to males. Furthermore, ChT exhibited a significant eccentricity by hemiretina by age interaction (*P* < 0.001) (Fig. 2). Across all of the nasal eccentricities, ChT increased with age from childhood to adulthood (*P* < 0.05). In contrast, a significant age-related thinning of the choroid was observed in the temporal perifovea, which was statistically significant from adolescence to adulthood (*P* = 0.021).

**Table 2.** Regional Changes in ChT, CVI, and Luminal and Stromal ChTs for Each Age Group

	Mean ± SEM			
	Children (*n* = 83)	Adolescents (*n* = 77)	Adults (*n* = 120)	*P*
**ChT (µm)**				
Eccentricity				
Fovea	343 ± 8	371 ± 8	378 ± 7	<0.001^a^
Parafovea	324 ± 8	353 ± 8	359 ± 7	0.144^b^
Perifovea	285 ± 7	306 ± 7	313 ± 6	
Hemiretina				
Temporal	341 ± 8	362 ± 8	347 ± 7	<0.001^a^
Nasal	293 ± 8	325 ± 8	353 ± 7	<0.001^b^
**CVI (%)**				
Eccentricity				
Fovea	63.9 ± 0.5	61.8 ± 0.6	57.6 ± 0.5	<0.001^a^
Parafovea	64.8 ± 0.5	63.3 ± 0.6	58.3 ± 0.5	0.256^b^
Perifovea	66.3 ± 0.5	65.0 ± 0.6	59.6 ± 0.5	
Hemiretina				
Temporal	63.8 ± 0.5	62.5 ± 0.6	58.1 ± 0.5	<0.001^a^
Nasal	66.2 ± 0.5	64.2 ± 0.6	58.9 ± 0.5	<0.001^b^
**Luminal ChT (µm)**				
Eccentricity				
Fovea	217 ± 5	229 ± 5	217 ± 4	<0.001^a^
Parafovea	208 ± 5	223 ± 5	208 ± 4	0.737^b^
Perifovea	186 ± 4	197 ± 4	185 ± 4	
Hemiretina				
Temporal	217 ± 5	226 ± 5	200 ± 4	<0.001^a^
Nasal	191 ± 4	207 ± 5	206 ± 4	<0.001^b^
**Stromal ChT (µm)**				
Eccentricity				
Fovea	126 ± 4	142 ± 4	161 ± 4	<0.001^a^
Parafovea	116 ± 4	130 ± 4	151 ± 3	0.014^b^
Perifovea	97 ± 3	106 ± 3	125 ± 3	
Hemiretina				
Temporal	125 ± 4	136 ± 4	145 ± 3	<0.001^a^
Nasal	101 ± 4	117 ± 4	146 ± 3	<0.001^b^

a *P* value for the main effect of eccentricity and hemiretina on choroidal parameter.

b *P* value for the interaction between eccentricity and hemiretina with age on choroidal parameter.

### Choroidal Vascularity Index 

**Figure 3.**
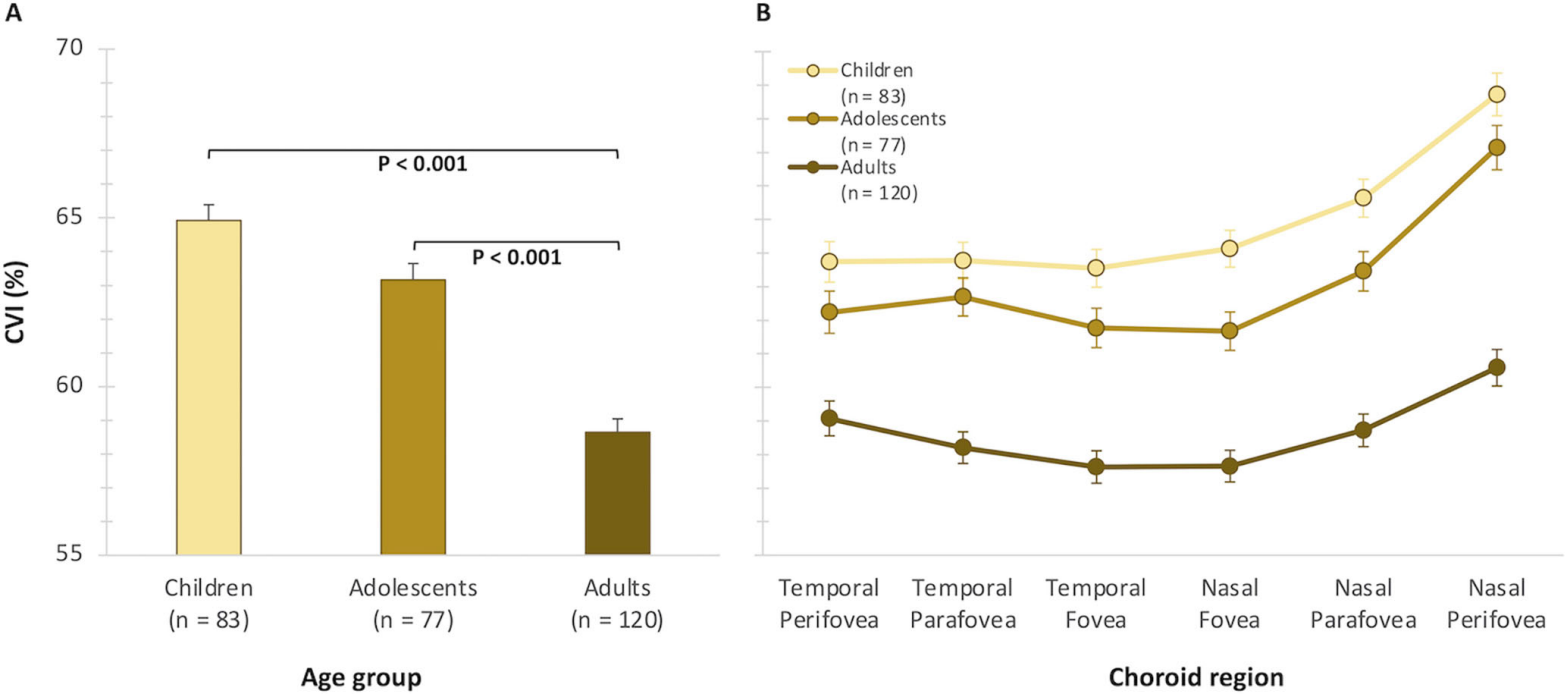
(**A**) Mean CVIs for each age group adjusted for gender. (**B**) Mean values for the CVI for each age group across different macular eccentricities and hemiretina. *Error bars* indicate the SEM.

The mean macular CVI was 61.8% ± 5.1%. The CVI was significantly associated with both gender and age. Overall, females exhibited a greater mean CVI (63.1% ± 0.36%) than males (61.5% ± 0.38%; *P* = 0.002). The CVI also reduced significantly from childhood to adulthood (*P* < 0.001) (Table 2; Fig. 3). The age-related changes in the CVI did not vary by gender (*P* = 0.519). Also, there was no significant effect of imaging time of day (*P* = 0.173). The CVI displayed significant topographical variation across both macular eccentricities and hemiretina (*P* < 0.001) (Table 2). The CVI was the lowest at the fovea (61.1% ± 0.29%) and increased progressively toward the parafovea (62.2% ± 0.28%) and perifovea (63.6% ± 0.29%). Significantly higher CVI values were seen in the nasal hemiretina (63.1% ± 0.28%) compared to the temporal hemiretina (61.4% ± 0.28%).

A significant hemiretina by age interaction was also observed, with the age-related decrease in the CVI being slightly greater in the nasal macula (−7.4% ± 0.7%) than the temporal macula (−5.6% ± 0.7%; *P* < 0.001). Furthermore, there was a statistically significant eccentricity by hemiretina by age group interaction (*P* = 0.002), with the decrease in CVI from children to adults being greater in the nasal than temporal eccentricities (Fig. 3B). All pairwise comparisons were significant (*P* < 0.05), except for the children versus adolescents age groups in the nasal eccentricities and children versus adolescents groups at all three levels of eccentricity in the temporal macular hemiretina (*P* > 0.05).

### Luminal and Stromal ChTs

**Figure 4.**
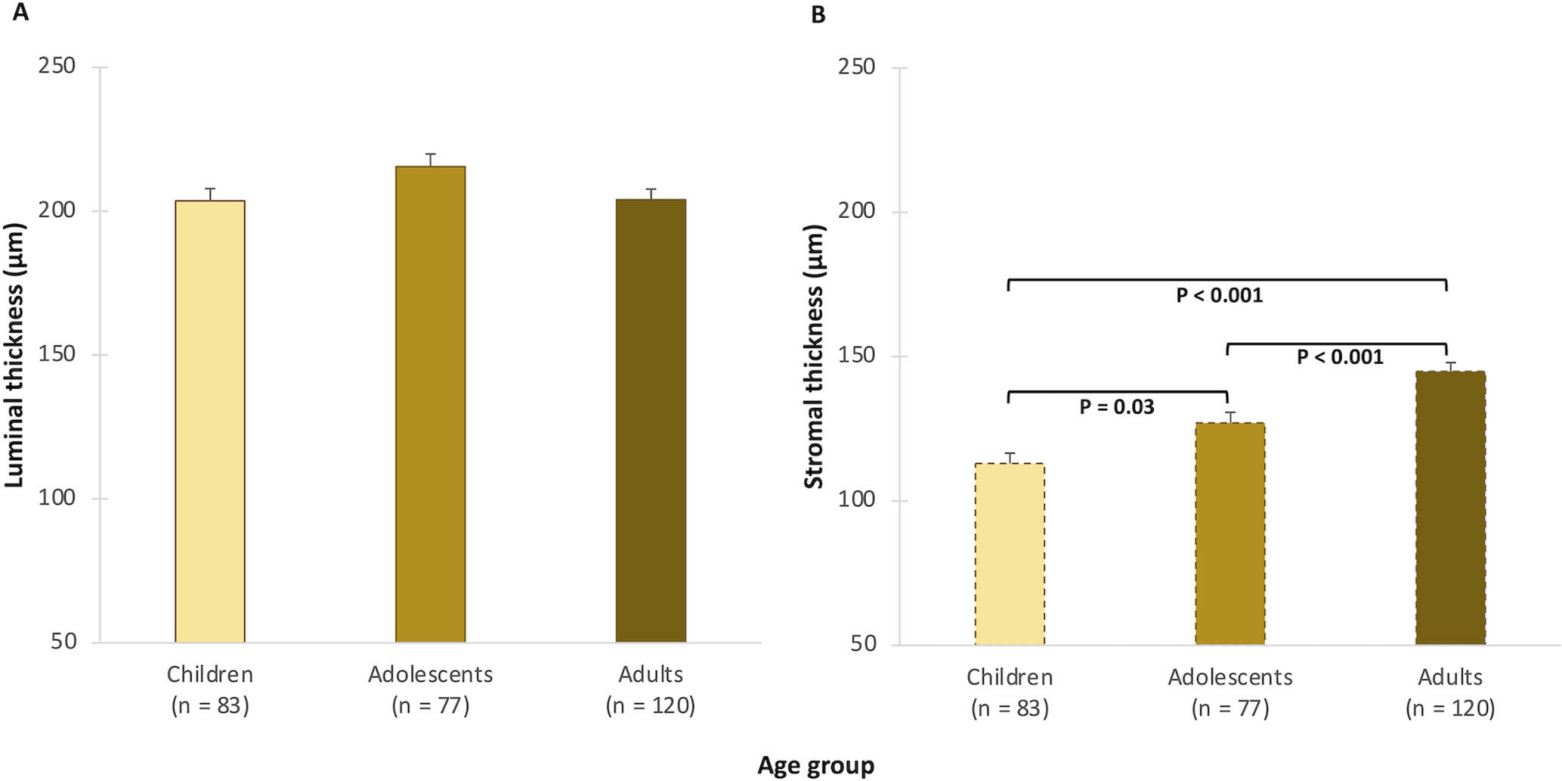
(**A**, **B**) Mean luminal ChT (**A**) and stromal ChT (**B**) for each age group adjusted for gender. *Error bars* indicate the SEM.

The mean luminal and stromal ChTs were 207 ± 39 µm and 130 ± 35 µm, respectively. Both luminal and stromal thicknesses did not vary significantly with gender or imaging time of day (*P* > 0.05). Overall, there was a statistically significant main effect of age on measures of stromal thickness (*P* < 0.001) but not luminal thickness (*P* = 0.068). Stromal thickness increased significantly from childhood to adulthood, with the thinnest profile seen in children (113 ± 4 µm), intermediate thickness profile in adolescents (126 ± 4 µm), and the thickest profile in adults (146 ± 3 µm). In contrast, a trend was observed for luminal thickness to increase from children (204 ± 4 µm) to adolescents (216 ± 5 µm) before declining in adults (203 ± 4 µm) (Fig. 4). These age-related changes in luminal and stromal thickness did not vary significantly by gender (*P* > 0.05).

**Figure 5.**
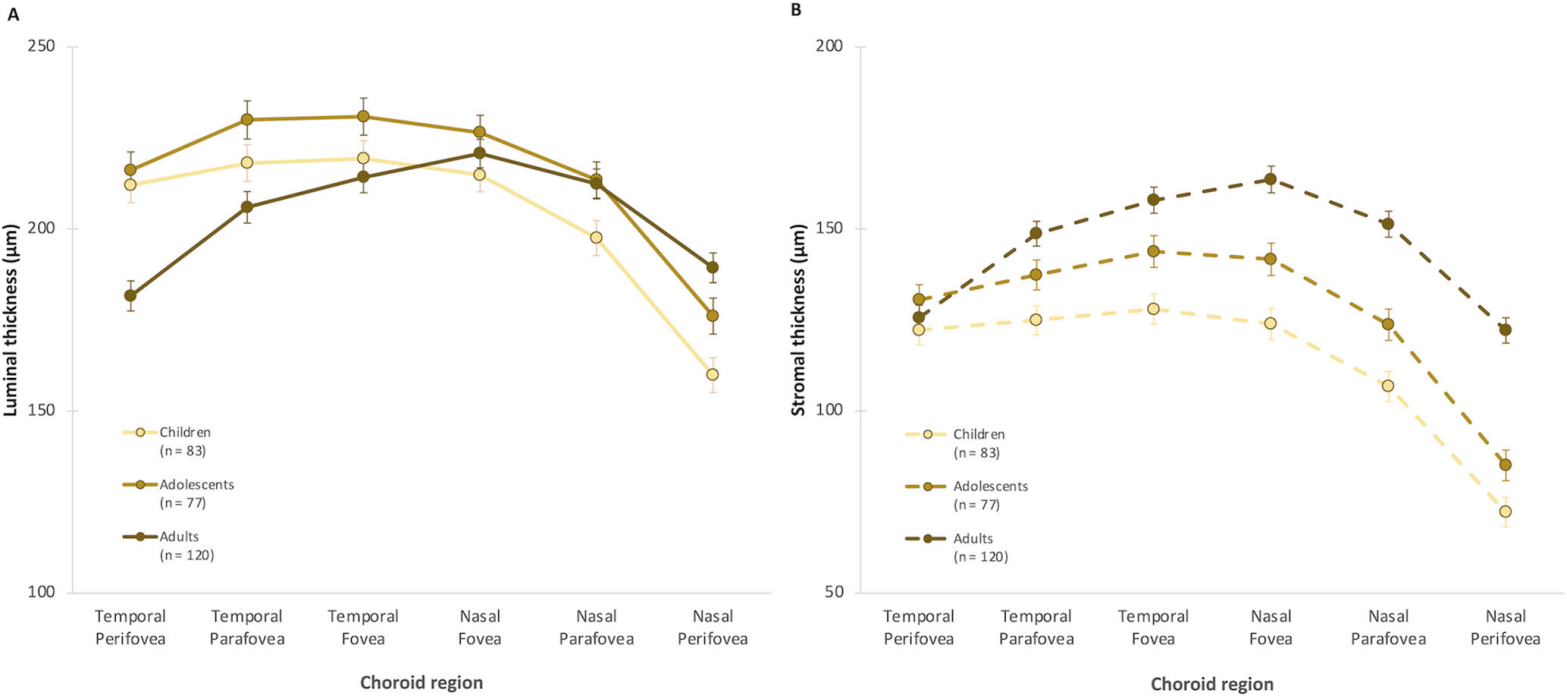
(**A**, **B**) Mean values for luminal thickness (**A**) and stromal thickness (**B**) for each age group across different macular eccentricities and hemiretina. Note the *y*-axis scales differ between the two graphs. *Error bars* indicate the SEM.

Statistically significant eccentricity- and hemiretinal-dependent variations were observed for both stromal and luminal ChT (*P* < 0.001) (Table 2). Both luminal and stromal ChT reduced significantly from the fovea toward the perifovea (*P* < 0.001) (Table 2). A statistically significant hemiretina by age interaction was observed for luminal and stromal ChT (*P* < 0.001 for both), although the nature of this effect varied between the two parameters (Fig. 5). The decline in luminal thickness from adolescents to adults was more pronounced in the temporal macula (−26 ± 7 µm; *P* ≤ 0.001), whereas no significant difference was observed in the nasal macula (*P* = 0.99). In contrast, the age-related increase in stromal thickness from children to adults was greater in the nasal macula (45 ± 5 µm; *P* < 0.001) than the temporal macula (21 ± 5 µm; *P* < 0.001). All pairwise comparisons among age groups were significant (*P* < 0.05) in the nasal macula.

The age-related changes in luminal thickness were consistent across various eccentricities (*P* = 0.89). However, a significant eccentricity by age interaction was observed in stromal thickness (*P* = 0.044), with an age-related increase in stromal thickness that was more pronounced in the fovea (change in children vs. adults, 35 ± 6 µm; *P* < 0.001) compared to the perifovea (change in children vs. adults, 28 ± 4 µm; *P* < 0.001).

Furthermore, there was a significant eccentricity by hemiretina by age interaction for both luminal and stromal thickness measurements (*P* < 0.001). Age-related changes in luminal thickness were most pronounced in the temporal perifovea, with a significant decline from children to adults and adolescents to adults (all pairwise comparisons, *P* < 0.001), but not from children to adolescents (pairwise comparison, *P* = 0.99). In contrast, the increase in stromal thickness from children to adults was greatest in the nasal eccentricities (*P* < 0.05 for all pairwise comparisons except children vs. adolescents in the nasal perifovea, where *P* = 0.88).

### Associations Among Choroidal Vascularity Parameters With Age, Axial Length, and ChT

**Figure 6.**
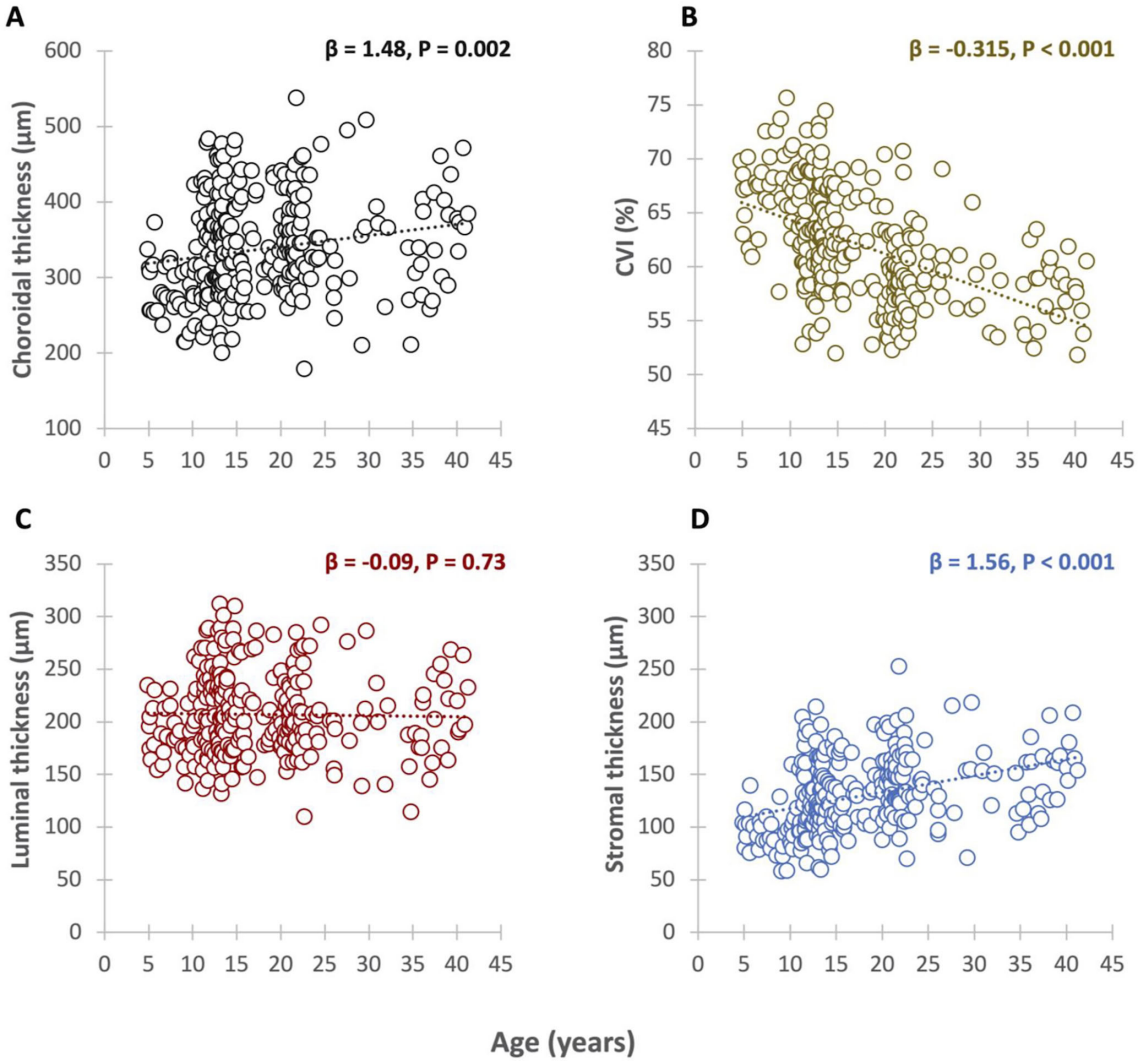
Univariate regression analysis between choroidal parameters and age. Showing choroidal thickness (**A**), CVI (**B**), Luminal thickness (**C**) and Stromal thickness (**D**).

Univariate linear regression analysis demonstrated a significant positive correlation between macular ChT (β = 1.48; *P* = 0.002) and stromal ChT (β = 1.56; *P* < 0.001) with age. Conversely, a significant decrease in CVI (β = −0.315; *P* < 0.001) was observed with age, whereas no significant association was found between the luminal thickness and age (β = −0.09; *P* = 0.73) (Fig. 6). The associations between age and the mean macular ChT (β = 1.7; 95% confidence interval [CI], 0.7–2.7), stromal ChT (β = 0.9; 95% CI, 0.7–1.1), and CVI (β = −0.29; 95% CI, −0.35 to −0.22) were also significant in the multivariate regression analysis that was adjusted for gender and imaging time of day (*P* < 0.001 for all β coefficients).

Comparisons of choroidal parameters with axial length in the univariate regression analysis showed a significant decrease in CVI with increasing axial length (β = −1.317; *P* = 0.002), but this association was not significant in the multivariate regression analysis (*P* = 0.123). In comparison, ChT, stromal thickness, and luminal thickness were not significantly correlated with axial length in this cohort of emmetropic eyes (*P* > 0.05 for all parameters in univariate and multivariate analysis).

ChT was significantly correlated with other choroidal parameters. The mean CVI decreased with increasing ChT, whereas stromal thickness and luminal thickness were positively correlated with ChT in both the univariate and multivariate analyses adjusted for gender and imaging time of day (*P* < 0.001 for all choroidal parameters).

## Discussion

This study found significant regional changes in vascularity and luminal and stromal thickness of the choroid with age. To our knowledge, this is the first study that has analyzed the regional changes in the macular choroidal vascularity index with physiological aging from childhood into adulthood among emmetropic participants. The mean macular ChT was found to increase significantly from childhood to adolescence, with no further change into adulthood. Supporting these findings, other population studies have found increases in ChT from early childhood to adolescence in emmetropic pediatric cohorts.^42^^–^^44^ Although our study only captured the age-related changes in ChT up to 40 years of age, multiple studies support an age-related decline in ChT in older adult populations, suggesting that ChT may peak between 10 and 20 years of age before decreasing into older adulthood.^27^^,^^45^^,^^46^ One study, however, found a thickening of the subfoveal choroid in young adults into their 20s.^47^ Notably, in the current study, ChT increased significantly with age only in the nasal macular hemiretina, leading to a reduced temporal–nasal asymmetry in ChT with age. However, the age-related changes were consistent across the macular eccentricities, signifying a decrease in ChT from fovea to perifovea across all age groups. Although the exact mechanisms underlying these hemiretinal regional changes is unclear, there is an established association between choroidal thinning and the physiological axial elongation of the eye with aging.^35^^,^^48^ The observed preferential thinning of the choroid in the temporal compared to nasal region may be linked to greater axial elongation in the temporal area, an effect that could potentially be caused by mechanical constraints in the nasal region associated with the optic nerve head.^42^ Further research is needed to confirm this hypothesis.

Our investigation revealed a significant decline in the CVI from childhood and adolescence to adulthood. This finding is consistent with a majority of previous studies describing a negative correlation between CVI and age.^12^^–^^16^^,^^18^^,^^19^^,^^21^^,^^49^^,^^50^ Characterizing this effect, our multivariate linear regression analysis revealed a decline in the CVI of 0.29% for every 1-year increase in age. Similar to ChT, the age-related decline observed with the CVI was consistent across the macular eccentricities but was more evident in the nasal than temporal macula.

We observed a significant increase in stromal thickness from childhood to adulthood, an effect that was most pronounced in the nasal macula. Previous studies have demonstrated that normal eye growth in childhood is accompanied by an increase in ChT.^42^^–^^44^ Our findings indicate that this physiological increase in ChT appears to be driven mainly by changes in stromal thickness. In keeping with these results, Ho et al.^25^ found that stromal thickness increased at approximately double the rate of luminal thickness over an 18-month period among a pediatric cohort. Correspondingly, the current study found the age-related decrease in the CVI to be greatest in the nasal compared to temporal macula. In contrast, there was a trend for luminal thickness to increase from childhood to adolescence and then decline into adulthood. In keeping with these results, Wakatsuki et al.^51^ found a decline in subfoveal luminal ChT with age in a population of adults between 21 and 85 years old. In our study, however, the decline in luminal thickness in adults was only evident in the temporal macula. Compared to our study, Wakatsuki et al.^51^ examined an older population, which may contribute to the more prominent luminal changes observed in their study. Collectively, our findings demonstrate that the age-related decline in CVI is driven primarily by an age-related thickening of the stromal choroid in the nasal macula and thinning of the luminal choroid in the temporal macula.

Significant region-dependent variations were observed among all choroidal parameters. In keeping with previous studies, ChT and luminal and stromal ChTs were greatest at the fovea with progressive thinning observed toward the perifovea.^3^^,^^8^^,^^52^^–^^54^ High metabolic demands of the retina at the fovea as the site of maximal cone photoreceptor density may contribute to the increased ChT in this region.^42^ In addition, choroidal thinning with increasing distance from the fovea was more pronounced in the nasal hemiretina, particularly in children and adolescents, for all three parameters. Thinning of the choroid nasal to the fovea could be multifactorial and explained by the anatomical location of the optic nerve, cilioretinal vessels, and posterior scleral foramen.^42^ Additionally, the main posterior watershed zone, representing the area in between two vascular regions supplied by the nasal and temporal ciliary arteries of the choroid, is situated between the nasal border of the optic disc and the fovea.^55^ The anatomical spatial arrangement of non-vascular smooth muscle cells in the outer choroid, which is particularly abundant at the macula and in the temporal compared to nasal choroid, may in part explain the thickening of stromal choroid in these areas.^13^^,^^56^

Consistent with findings of prior studies,^4^^,^^8^^,^^14^^,^^57^^,^^58^ we found that the mean luminal thickness was greater than the mean stromal thickness across the macula. Reflecting this overall higher average luminal choroidal area, the mean CVI across all subjects in our study was 61.8% ± 5.1%, which is largely in agreement with normative ranges reported in previous studies.^4^^,^^5^^,^^16^^,^^20^^,^^24^

Inconsistent reports of topographical variations in the CVI exist within the literature. We found that the CVI was lowest at the fovea and increased toward the parafovea and perifovea. This has been validated in a recent large-scale study of 5864 children in China, with different refractive states demonstrating lowest CVI values at the fovea.^59^ Another study in healthy young adults reported lowest CVI values at the fovea which increased toward the periphery, an effect that was sustained across extramacular regions that were not examined in our study.^8^ A greater proportion of large choroidal vessels with increasing distance from the fovea may explain the increase in the CVI with eccentricity.^49^

We also found that the mean CVI was significantly higher in the nasal compared to temporal macula. This regional variation across the horizontal meridian is in keeping with previous reports.^8^^,^^49^^,^^59^ In a postmortem study of eight whole retina, Curcio et al.^60^ found that cone receptor density was 40% to 45% higher in the nasal compared to temporal retina. Given that choroidal properties are intimately connected to photoreceptor demand, this spatial variation in photoreceptor density would likely contribute to the higher CVI values obtained in the nasal macular hemiretina.

Although our findings demonstrated luminal thinning with increasing macula eccentricity, the associated increase in the CVI suggests that there is a disparity in the rate of luminal thinning compared to stromal thinning. As the CVI is the ratio of luminal thickness to total ChT, our data indicate that the degree of luminal thinning with increasing eccentricity (−31.9 ± 1.4 µm) is less pronounced compared to its stromal counterpart (−33.5 ± 1.3 µm). Correspondingly, the rate of luminal thinning between temporal and nasal macula hemiretina (−13.1 ± 1.1 µm) was reduced in contrast to stromal thinning (−14.3 ± 1.0 µm). Collectively, this suggests that topographical changes in macular ChT and the CVI in our cohort was primarily a function of changes in stromal thickness. Although mechanisms underlying the age-related regional changes in luminal and choroidal thickness are beyond the scope of this study, alterations in stromal components including connective tissue, non-vascular smooth muscles, and intrinsic choroidal neurons might contribute to the observed thickening of the stromal layer with age in the nasal area.^56^ Additionally, the greater age-related thinning of the luminal choroid in the temporal region may predispose the temporal retina to pathologies linked to reduced choroidal perfusion such as age-related macular degeneration. It is also suggested that choroidal thinning may be more pronounced in the temporal region in age-related macular degeneration.^61^ More research is required to explore the potential implications of findings from this study in the pathophysiology of ocular diseases.

Further examining the relationship between ChT and other choroidal parameters, univariate and multivariate linear regression analyses revealed a significant positive relationship between ChT and luminal and stromal thickness. Conversely, the CVI exhibited a negative correlation with ChT, paralleling the increase in the CVI despite declining ChT with increasing eccentricity.

Overall, females exhibited a statistically significant higher mean CVI (63.0% ± 0.36%) than males (61.5% ± 0.38%), but ChT, luminal thickness, and stromal thickness did not vary significantly between the genders. This is in contrast to a majority of previous studies that have not observed a statistically significant difference in the CVI between the sexes.^12^^,^^13^^,^^19^^,^^62^^,^^63^

Axial length measurements were reflective of an emmetropic cohort with a mean of 23.35 ± 0.71mm. Within this demographic, multivariate linear regression analysis demonstrated no significant associations between any of the choroidal parameters and axial length, adjusting for age, gender, and imaging time. Prior studies have demonstrated thinning of subfoveal ChT with increasing axial length specifically in myopic subjects,^6^^,^^64^ whereas data on the CVI are inconsistent.^8^^,^^21^^,^^24^^,^^65^^–^^72^ Importance is given to understanding the relationship between choroidal vascularity and axial length, as both are implicated in the pathogenesis of degenerative myopia.^73^ Our findings are consistent with those of Breher et al.,^52^ who also found no significant correlation between the CVI and axial length in healthy emmetropic eyes. Although such data cannot be extrapolated to myopic eyes, they demonstrate that choroidal parameters appear to be independent of axial length in emmetropes.

### Strengths, Limitations, and Future Directions

This study is strengthened by the use of semi-automatic segmentation and automatic binarization of OCT images with custom-written software based on deep learning algorithms optimized for CVI analysis and that have been previously validated in robust cohort studies.^39^^–^^41^ It is worth noting, however, that although inter-cohort CVI measures are generally agreeable, validation of the CVI with reference to a predetermined gold standard such as histology is yet to be established.^13^ A considerable sample size of 280 emmetropic eyes were examined in this study while controlling for the potential effect of confounding factors, including smoking, pharmacological agents, optical defocus, and the influence of refractive error on choroidal parameters. Further research is required to define age-related changes in myopic and hyperopic eyes to understand the interaction between refractive error development and aging effects. Additionally, factors such as body mass index, hydration, blood pressure, and caffeine intake in the younger participants may have confounded measurements of choroidal thickness and vascularity, and future studies are required to explore whether these factors interact with age-related changes in choroidal parameters.

Data availability for this study was limited to subjects between the ages of 5 and 41 years old. Although there were sufficiently large numbers to characterize changes among the three specified age groups (children, adolescents, and adults), future studies are required to examine choroidal parameters in infants, young children, and older populations. Overall, there is a paucity of data on choroidal parameters in infants and young children, restricted by the feasibility of performing reliable OCT imaging in this age group.^74^ The advent of handheld OCT prototypes introduces a potential avenue for further research in this area.^75^ Beyond the fourth decade of life, previous studies primarily support a sustained age-related decline in the CVI; however, further studies in spatial variations with age in older demographics are required.^13^^–^^16^^,^^19^

In this study, OCT imaging was conducted with only horizontal scans across a 5-mm macula region, whereas further evaluation with scans across the vertical meridian, as well as with increasing peripheral eccentricity, would be valuable. Furthermore, volumetric scans or combining a binarization method with OCT angiography would enable simultaneous sub-analyses of choriocapillaris density and choroidal vascular area.^68^^,^^76^

## Conclusions

In conclusion, quantifying choroidal vascularity can provide valuable information on ocular status in health and disease. To date, there is a paucity of normative data for the CVI and a further absence of information regarding spatial variations in the CVI during physiological aging. In this study of healthy emmetropic eyes, increasing age from childhood into adulthood was associated with a significant region-dependent decline in the CVI. In the nasal macula, the age-related reduction in the CVI was primarily driven by thickening of the stromal choroid. Comparatively, thinning of the luminal choroid in the temporal macula was responsible for the observed decline in the CVI in this region. Temporal variations in the CVI were consistent across genders, supporting its utility as a robust clinical biomarker. These age-related changes in the CVI provide new insights into the morphology of the choroid and its possible role in the pathogenesis of ocular diseases. Findings from this study may explain why some chorioretinal diseases have certain spatial or age-associated predilections, providing a promising framework for further research into the CVI and its integration into clinical practice.
